# Hostility Toward Baby Boomers on TikTok

**DOI:** 10.1093/geront/gnac020

**Published:** 2022-02-01

**Authors:** Reuben Ng, Nicole Indran

**Affiliations:** Lee Kuan Yew School of Public Policy, National University of Singapore, Singapore, Singapore; Lloyd’s Register Foundation Institute for the Public Understanding of Risk, National University of Singapore, Singapore, Singapore; Lee Kuan Yew School of Public Policy, National University of Singapore, Singapore, Singapore

**Keywords:** Content analysis, Generational stereotypes, Intergenerational tension, TikTok, Videos

## Abstract

**Background and Objectives:**

The recent entry of the hashtag #OkBoomer into social media vernacular underscores the collective frustration of younger people with a group whose views they find increasingly incompatible with theirs. Most social media analyses in gerontology focus on the content on Twitter and Facebook, with content on TikTok virtually unexplored. Given the burgeoning popularity of TikTok among younger people, we assess the content of TikTok videos with the hashtags #OkBoomer or #Boomer to distill the undercurrents of hostility expressed by younger people toward Baby Boomers.

**Research Design and Methods:**

We collated TikTok videos (*N* = 332) with the hashtags #OkBoomer or #Boomer, which received over 5.4 billion views. Both inductive and deductive approaches guided the qualitative content analysis of the videos.

**Results:**

Five themes emerged. Most videos (79%) described “Negative Encounters with Baby Boomers” (Theme 1); 58% were about “Conflicting Values/Beliefs between Baby Boomers and Younger People” (Theme 2); 39% were about “Baby Boomers Antagonizing Younger Generations” (Theme 3); 22% of the videos made references to the “Karen Meme” (Theme 4); and 7% bemoaned the existence of a “Wealth Gap” between Baby Boomers and younger people (Theme 5).

**Discussion and Implications:**

Findings reveal that the usage of the hashtags #OkBoomer and #Boomer is highly nuanced, at times explicitly ageist, and at others, emblematic of a phenomenon far more complex than ageism. There is a need to leverage social media as a space to foster interaction between older and younger people. Society is ultimately well served by intergenerational interaction.

With the effects of population aging unfolding, intergenerational solidarity has slowly become a priority concern in many societies (Pinazo-Hernandis & Tompkins, 2010). However, since the coronavirus disease 2019 (COVID-19) outbreak, scholars have argued that there has been a surge in both ageism and intergenerational tension (Ayalon, 2020; Meisner, 2020). The frequent depiction of older adults as a high-risk cohort for COVID-19 has led to the worrying trivialization of the virus as the problem of older adults, with some crassly referring to it as a “Boomer Remover” (Fraser et al., 2020; Lichtenstein, 2020; Meisner, 2020). During the peak of the pandemic, young people were criticized for flouting safe distancing rules (Casey, 2020), while others pointed their fingers at older people for failing to heed health warnings (Pariser, 2020).

Concerns that there may be a yawning chasm between younger and older people, however, predate the pandemic. While “Baby Boomer” was merely a form of demographic classification in the past, today the term serves as a problematic and imprecise shorthand used to denigrate older adults who fall within a particular age bracket. Bristow (2019) explicates the notion of “Boomer blaming” to refer to the incendiary rhetoric used to describe Baby Boomers in the media, where numerous articles vilify them for having “wrecked the economy” and “stolen their children’s future” (Bristow, 2019). Similarly, the emergence of the expression “Ok, Boomer” suggests that the label has become a pejorative used to denounce values and beliefs seen as stymying progressive ideas (Gonyea & Hudson, 2020; Lyons, 2020).

The benefits of intergenerational interaction have been well documented. Not only do interactions between different generations offer a healthy mechanism for the mutual exchange of knowledge, skills, and values, they also improve the social skills of both younger and older groups (Boger & Mercer, 2017). As members of the Baby Boomer cohort enter later life, it is vital that they age well in all facets of life, particularly on the social front. Maintaining meaningful social relationships and feeling valued have both been widely acknowledged to be key elements of aging well (Cortellesi & Kernan, 2016; Leist, 2013). It is thus imperative to ensure that perceived differences across generational lines do not devolve into ageist practices. To reduce intergenerational animosity, an understanding of prevailing sentiments toward older adults is necessary. Our study provides insight into this topic by analyzing TikTok videos with the hashtags #Boomer and #OkBoomer. Specifically, we analyze the undercurrents of hostility expressed by younger people toward older adults from the Baby Boomer generation.

The concept of generations has origins in sociology. Mannheim (1952) distinguished three features that define a generation. First, a generation is a location that unites individuals born in the same period. Second, a generation is an actuality that shares certain experiences that in turn predispose individuals to particular ways of thinking. Third, within an actuality are generational units or groups of individuals who develop distinct responses to the same sociohistorical conditions. From this perspective, generations can be viewed as complex social groups embedded within particular social, cultural, and historical contexts, bound by a sense of collective consciousness (Lyons & Kuron, 2014; Mannheim, 1952).

Today, generational markers typically function as heuristics to group people into various demographic cohorts. Individuals born between 1946 and 1964 are known as “Baby Boomers.” Succeeding the Baby Boomers is “Generation X,” which comprises individuals born between 1965 and 1980. Members of “Generation Y,” commonly known as “Millennials,” are generally defined as born between 1981 and 1996. “Generation Z” is composed of those born from 1997 to 2012 (Dimock, 2019).

Recently, there have been calls by social scientists to retire the use of generational labels (Cohen, 2021; Pinsker, 2021). While analytically convenient, generational labels are set on arbitrarily drawn boundaries. That the exact parameters chosen to demarcate generational cutoff points lack consistency across different sources reiterates the fuzziness of the concept (Campbell et al., 2017; Cohen, 2021). More disconcertingly, generational labels have been used to perpetuate the myth that members of any given generation share the same characteristics (Cohen, 2021; Pinsker, 2021). Such a totalizing view of generations, also known as “generationalism” (Purhonen, 2016; Rauvola et al., 2018), fuels the formation of inaccurate stereotypes. Each generational label carries certain meanings that obfuscate major differences in outlooks and experiences. Millennials are frequently criticized for being “snowflakes,” Baby Boomers for being self-centered, and Generation Z for being emotionally fragile (Lyons, 2020). Compounding the issue is the fact that generational categories are often evoked in public discourse not only to draw comparisons across different cohorts, but also to pit one generation against another (Lyons, 2020).

Social identity theory posits that individuals’ self-concepts are shaped by the social categories that they see themselves belonging to (Tajfel, 1974). According to this theory, people classify themselves and others based on perceived similarities and differences, a process that may lead to the preference of an ingroup over an outgroup (Tajfel, 1974). Intergroup bias may function as a result of favoritism toward the ingroup or hostility toward the outgroup (Branscombe et al., 1999; Tajfel, 1974). As individuals form ingroups and outgroups on the basis of generational identity, intergenerational conflict may ensue (Sipocz et al., 2021). When left unchecked, generationalism may lead to ageism—a form of stereotyping, prejudice, and discrimination on the grounds of age (Costanza & Finkelstein, 2015; Ng & Indran, 2021c; Ng & Lim, 2021; Rudolph & Zacher, 2020).

Our decision to analyze videos on TikTok was motivated by three reasons. First, the application has received scant attention among researchers in the field of gerontology. Second, since debuting in 2016, TikTok has secured a strong foothold in the social media landscape. Its rapid ascent was propelled further during the pandemic with media consumption hitting an all-time high (Fishwick, 2020), rendering it a powerful tool in shaping public opinion. Third, TikTok, more so than any other social media application, is known to be inhabited predominantly by younger people (Muliadi, 2020), making the platform a potentially rich source of insight.

Our study is significant on two levels. On a conceptual level, this is one of the first known studies exploring hostility toward Baby Boomers using videos, particularly on a platform widely used by younger people. To our knowledge, only one other study has analyzed videos about the Baby Boomer generation on TikTok (Zeng & Abidin, 2021), though Zeng’s and Abidin’s study was more on how younger people draw on meme and video cultures to navigate intergenerational politics. Additionally, most gerontological studies that look at social media examine content on Twitter (Jimenez-Sotomayor et al., 2020; Sipocz et al., 2021; Skipper & Rose, 2021) and Facebook (Levy et al., 2014), with content on TikTok unexplored. On a practical level, our study furnishes an overview of younger people’s attitudes toward Baby Boomers—an essential step in managing intergenerational discord.

To understand the factors fueling hostility toward Baby Boomers, we pose the following research question: What are the themes present in videos where younger people exhibit hostile attitudes toward Baby Boomers? To address this question, we adopted both inductive and deductive approaches to qualitatively analyze TikTok videos catalogued under either the hashtag #Boomer or #OkBoomer.

We considered the following complexities in undertaking this unprecedented study. First, whether the individuals uploading the TikTok videos are cognizant of the range of birth years used to define the Baby Boomer generation is unknown. However, because most people tend to be unaware of which generation they belong to (Pinsker, 2021), it is plausible that some content creators employ “Baby Boomer” as a blanket term to refer to individuals whom they perceive as old, although what qualifies as old is unclear and subject to variation. Second, throughout this study, we use generational labels to refer to specific generations. In employing these terms, our intention is not to homogenize members of each generation but rather to ensure consistency with mainstream discourse on the subject (Campbell et al., 2017). Third, we use the terms “young” and “younger” to refer to Millennials and members of Generation Z. We use “old” and “older” to refer to individuals aged between 57 and 75, in keeping with the most frequently defined age range of Baby Boomers.

## Method

Similar to earlier work (Herrick et al., 2020), we created a new TikTok account to collate the videos. We did this to minimize bias because videos on the application are sorted based on a complex algorithm that takes into account the popularity of the post (measured by views, likes, comments, and shares), the popularity of the creator (measured by followers and engagement), any previous content that was engaged with, and the geographical location of the device used to access the application (Herrick et al., 2020). We did not engage with any content prior to the data collection phase in order to guarantee a common user’s experience in exploring the application (Herrick et al., 2020).

This study analyzed videos appended with the hashtags #Boomer and #OkBoomer. All publicly available videos that appeared under the hashtags were compiled. There were 988 videos tagged #Boomer and 949 tagged #OkBoomer that received over 5.4 billion views. We considered but did not include other hashtags (e.g., #BoomerRemover) as they did not gain as much traction on TikTok.

**Figure 1.**
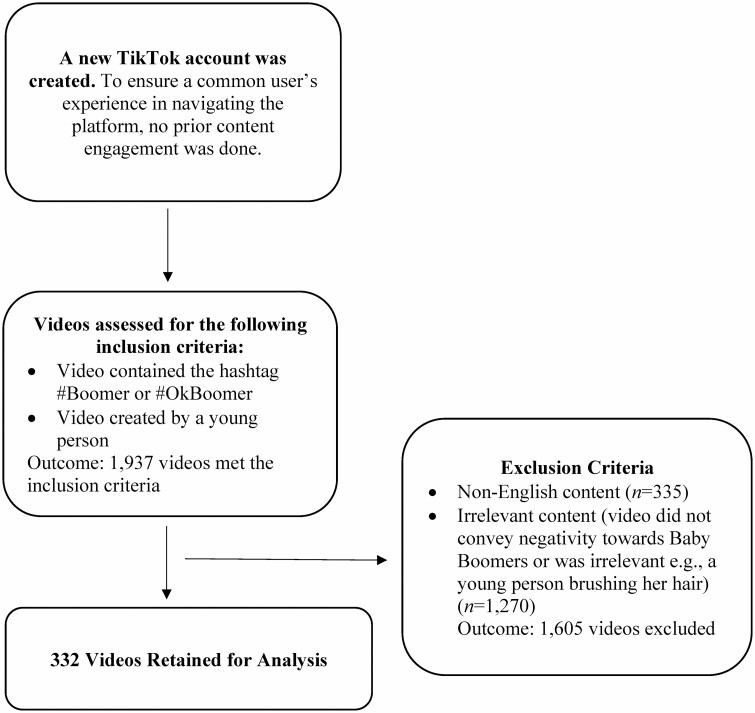
Process of and criteria for collating TikTok videos created by younger people about Baby Boomers.

Videos were selected based on the following inclusion criteria: (a) video is created by a younger person. At this point, it is worth noting that videos on TikTok often feature the account holder. We excluded videos created by older adults as the focus of the study was on the perceptions that young people have of older adults; (b) video is in English; (c) content must be relevant to the hashtag, that is, video should be about or refer to older adults; and (d) video contains negative content regarding Baby Boomers. All videos had a maximum length of 1 min as mandated by the platform during the study period. The entire process culminated in 332 publicly available videos. Figure 1 provides a flowchart of the data collection process.

To meet the first inclusion criterion, we processed the video frames via Microsoft Azure’s Face Recognition Platform (Face API: An AI service that analyzes faces in images, 2021). This software extracts information regarding age and has been used in earlier work related to TikTok (Fiallos et al., 2021; Serrano et al., 2020). In this study, “young” and “younger” referred to Millennials and members of Generation Z. The maximum age of a “younger” user was 40—the age of the oldest Millennial in 2021. We included content created by this group. “Old” and “older” were used to refer to creators aged 57—the age of the youngest Baby Boomer in 2021—and older. We excluded content created by this group. Though we acknowledge that the software is not always accurate, it nonetheless provided a more objective and consistent method of retrieving information about age.

### Video Content Coding

In line with prior research (Sipocz et al., 2021), the coding rubric was developed through both inductive and deductive modes of reasoning (Armat et al., 2018). In inductive content analyses, codes are derived directly from the data (Hsieh & Shannon, 2005). Meanwhile, analyses led by a directed or deductive approach begin with the identification of an initial set of codes based on prior literature (Hsieh & Shannon, 2005). We adopted both inductive and deductive approaches to ensure that certain pertinent assumptions guided the analysis while also mindful that new categories would emerge inductively (Armat et al., 2018).

To create a preliminary codebook, we first identified a set of categories based on past literature regarding intergenerational tension and generational stereotypes (Lyons & Kuron, 2014; Sipocz et al., 2021). The content analysis was subsequently conducted in several stages, with each video viewed twice by two researchers trained in gerontology to ensure familiarity with and immersion in the data (Hsieh & Shannon, 2005). The purpose of the first viewing was to confirm the validity of the initial set of categories, as well as to generate codes systematically across the entire data set. Each researcher modified the codebook independently until all variables were refined and clearly defined. During the first viewing, a new category was added whenever a video featured a particular attribute that could not be suitably coded into any of the existing categories and which was recurrent in the data. During the second viewing, the two coders had frequent discussions, where any discrepancies were reviewed and adjudicated to ensure rigor in the analysis. At this point, the two coders discussed what the codes meant, ensured the relevance of the codes to the research question, and identified areas of significant overlap in order to finalize the coding rubric. The percentage agreement between the two raters was 97.5% with a weighted Cohen’s kappa of 0.94 (*p* < .001), indicating high interrater reliability.

In total, 34 codes were generated. We made connections across these codes to sieve out probable themes in the data. We then refined the themes to ensure as much internal homogeneity and external heterogeneity as possible while recognizing that categories in a qualitative content analysis need not be mutually exclusive (Bengtsson, 2016; Sipocz et al., 2021; Tesch, 1990). A total of five themes emerged from the whole process, and the frequency of each theme was identified postanalysis. The themes are summarized in Table 1.

**Table 1.** Description of Themes of TikTok Videos Created by Younger People About Baby Boomers

Theme	Description	%
Negative encounters with Baby Boomers	User recounts a negative experience with an individual who happened to be older.	79
Conflicting values/beliefs between Baby Boomers and younger people	User describes Baby Boomers as having beliefs—religious, political, social, personal, etc.—that contradict those of younger people.	58
Baby Boomers antagonizing younger generations	User shares about being antagonized by Baby Boomers on the basis of the former’s generational membership as a younger person.	39
The “Karen” meme	User makes references to the “Karen” meme.	22
Wealth gap	User bemoans the existence of a generational wealth gap between Baby Boomers and younger generations.	7

## Results

### Summary of Insights From Content Analysis of TikTok Videos

Five themes emerged from a content analysis of 332 TikTok videos featuring displays of hostility toward Baby Boomers. Theme 1 “Negative Encounters with Baby Boomers” formed the majority of the videos (79%, *N* = 262). Examples of videos belonging to this theme involve the user recounting or recording a negative experience with an individual who happened to be older. Theme 2 “Conflicting Values/Beliefs between Baby Boomers and Younger People” appeared in 58% of the videos (*N* = 192). Videos under this theme include the user describing Baby Boomers as holding beliefs—religious, political, social, personal, etc.—that contradict those of younger people. Theme 3 “Baby Boomers Antagonizing Younger Generations” was in 39% of the videos (*N* = 130). Uploads under this theme include users sharing about being antagonized by Baby Boomers on the basis of the former’s generational membership as a younger person. Theme 4 revolves around references to the “Karen” meme (e.g., user refers to a Baby Boomer as a “Karen”) and was present in 22% of the videos (*N* = 72). Theme 5 “Wealth Gap,” where the user bemoans the existence of a wealth gap between Baby Boomers and younger generations, was found in 7% of the videos (see Table 1 for a summary).

### Negative Encounters With Baby Boomers (Theme 1; 79%)

Videos filed under this theme featured younger people narrating negative encounters with individuals who happened to be older. For example, one user described feeling patronized by an “elderly woman” who told her she was “pretty for a crippled girl” and that “sick people are some of the happiest people.” Another TikToker recalled a run-in with an older man, who upon noticing her t-shirt was emblazoned with the phrase “Be a nice human,” remarked, “When I grew up, we were allowed to hate people, and I think that’s okay.”

Other examples include encounters with Baby Boomers who would persistently stare or yell at others in public. For instance, a younger person documented his confrontation with an older individual who did not wear a mask despite being in a store which mandated mask-wearing. In this video, the young person called the older man out for exhibiting selfish behavior, to which the older man retorted, “You need to shut up and leave me alone. Walk away, do it now.”

### Conflicting Values/Beliefs Between Baby Boomers and Younger People (Theme 2; 58%)

In many of these videos, “Boomer” was used as a catch-all term to eschew behaviors and beliefs thought to be common to the Baby Boomer generation, such as those related to racism, homophobia, sexism, conservatism, religious extremism, the denial of climate change, and more recently, the refusal to wear masks. One video took on the style of a recipe with a text overlay indicating “How God made Boomers.” The TikToker prefaced the clip by saying, “Today we’ll be making Boomers. Ew. First, let’s make them hate the gays […] Let’s make their favorite channel Fox News. Let’s make their main source of happiness ‘minion’ memes on Facebook. Let’s make their favorite catchphrase ‘How do you work this?’ and their second favorite ‘When I was growing up’. Two shots of being a ‘Karen’ or a ‘Chad’, two shots of reposting religious quotes on Facebook.”

Another video featured a self-proclaimed Millennial delivering a tirade at Baby Boomers both for criticizing younger people as well as for possessing opinions believed to contradict the more progressive ideologies of younger people. In this clip, the TikToker raged, “Do y’all just not remember 2010 on when all anyone had to do was [expletive deleted] on the millennial generation?” Every news outlet, every magazine, Buzzfeed, all they had to talk about was like [*sic*], “Oh they’re the ‘me’ generation with their Instagrams and their narcissism and avocado toasts. All we did was flip that exact script. But instead of Instagram, it was systemic racism. Instead of avocado toast, it was environmental destruction for profit and instead of narcissism, it was late-stage capitalism. I’m sorry if turning the mirror on you hit it a little different.”

In some posts, differences in political beliefs led young people to make derogatory comments about older adults’ age-related vulnerabilities. One user juxtaposed a reenactment of a vibrant discussion with her schoolmates about how society “needs progressive climate, economic and social policies” against a reenactment of herself “explaining to conservative Boomers that Joe Biden isn’t a communist even though a Facebook post told them so.” She spoke in a slow and infantilizing tone in the latter reenactment, as reinforced by her use of a nursery rhyme as an audio clip. Similarly, numerous videos featured an audio clip with the lyrics “[...] sit back in your rocking chair, I don’t wanna see your white hair, voted for that cheeto puff (a reference to President Trump). Oh, you hate us? That’s tough”.

One user contrasted his interaction with a Baby Boomer before and during the COVID-19 pandemic. The first scene featured him before the pandemic in January 2020, imploring everyone—on behalf of teenagers—to “start recycling and stop polluting the earth” because “it’s dying.” He donned a mask of an older person in the subsequent scene and responded with a “Sorry, I missed the part where that is my problem.” The video then transitioned into another scene set during the pandemic, in which he, acting as a Baby Boomer, pleaded with teenagers to stay home in order not to spread the virus to him. The user then superimposed himself into a nightclub with a text sticker reading, “Teenagers hanging out with friends and spreading corona [*sic*],” before acting as the same Baby Boomer suffering in bed with the text sticker indicating “Boomers getting infected and dying.”

A significant number of videos looked at how Baby Boomers and younger people clash in their attitudes toward sexuality. One TikToker sardonically roleplayed as a prudish, straitlaced Baby Boomer who expressed disapproval of her decision to wear “fishnets” (i.e., a type of hosiery). This video was accompanied by an audio track with the lyrics “Somebody come fetch her, she’s performing like a harlot. Somebody come get her, she’s behaving like a wretch.”

Other relevant clips include exchanges with Baby Boomers whom young people perceived as stifling their desire for creativity and self-expression. For example, one individual reenacted an encounter with “some Boomer customer” who questioned her decision to dye her hair green: “Woah, green hair! Did you do that on purpose?” Similarly, “ripped jeans” were a recurring motif in many videos, with younger people frustrated with Baby Boomers for laughing at or deriding them for their sartorial choices.

### Baby Boomers Antagonizing Younger Generations (Theme 3; 39%)

In these videos, younger people felt that they were often lambasted by virtue of their being Millennials or members of Generation Z. These videos were retaliatory in nature, with younger people feeling unfairly maligned by Baby Boomers. One TikToker reenacted a conversation during which he was berated by his parent. “That’s the thing with you kids. Y’all are ungrateful and disrespectful. I had to raise you, I had to clean you. I brought you into this world and I will definitely take you out.” The TikToker’s rejoinder was an acerbic “You think I [expletive deleted] asked for this [expletive deleted]?”

Many posts were about Baby Boomers making pointed remarks about young people’s addiction to their phones. One such post featured a younger person acting like an older adult. “Everyone in the younger generation is just so anxious nowadays. I really think it’s all that screen time.” A user in another clip also roleplayed as an older person saying, “I’m going to unplug your [expletive deleted] phone,” to which the young person clapped back, “I’m going to unplug your [expletive deleted] life support!”

Some videos were essentially commentaries about the tendency of older adults to hyperbolize the struggles they had in the past in order to position themselves as superior to younger generations or to guilt-trip them. This is best epitomized by a clip taken from a mobile game called Temple Run, in which the player, an explorer, traverses various obstacles while fleeing enemies. The video contained a text overlay saying, “How Boomers swear they got to school” and was supplemented with the caption, “My grandpa ran 20 miles uphill barefoot on dirt road with potholes and was never late to class.”

In one post, a self-identified 26-year-old reenacted a conversation with her “Boomer uncle” about being mired in college debt. In this clip, she dramatized the condescending way in which he dismissed her struggles: “You’re just too young to understand. You need more life experience […] Back in my day, I could get a Coca Cola for two cents and I could go to college for six dollars, so I think you just need to work harder.”

### The “Karen” Meme (Theme 4; 22%)

A common thread running through videos referencing the “Karen” meme has women displaying behaviors considered to be hostile and irrational. “Karen” behavior in most of these uploads included mistreating staff in the service line or demanding to “speak to the manager” when a certain product was out of stock. One video involved a TikToker ranting about an older lady who screamed at an employee at Starbucks because she was given the wrong order, an act which he labeled as a “real [expletive deleted] Karen move.” In another video, a user recounted a skirmish with a “Karen” who described a store as “ungodly” and “abominable” as she took issue with the fact that this store had set up an aisle displaying merchandise related to the gay community during Lesbian, Gay, Bisexual, Transgender, and Queer Pride Month.

### Wealth Gap (Theme 5; 7%)

Resentment toward Baby Boomers in uploads related to this theme stemmed from the belief that members of this generation have hoarded an enormous share of societal resources, bequeathed to younger generations a broken economy, and dismissed the younger generation’s struggles in securing a job. To enumerate various examples of the economic struggles experienced by Millennials and members of Generation Z, one user superimposed text overlays on his videos airing the following grievances: “I can’t afford rent,” “My children are starving,” “Inflation is insane,” “The housing market is against everyone,” and “I’m working 60 hours a week.” In this same clip, the user reenacted dismissive quips presumably uttered by Baby Boomers such as “I paid for college with a job at the grocery store,” “I bought a house at 20 years old,” “Get a better paying job,” “If you work hard [*sic*] you’ll be fine,” and “Stop asking for handouts.” Other posts include young people hitting back at older people for belittling their struggles with statistics showing that while “average college debt” had skyrocketed, “average graduate starting pay” had actually dropped.

## Discussion

When the hashtag #OkBoomer first went viral in 2019, many were quick to brand it as an ageist slur (Fabris, 2019). However, defenders of the phrase have since maintained that the phrase problematizes antiquated mindsets rather than old age per se (Fabris, 2019). Our results provide evidence that usage of the term is highly nuanced, at times explicitly ageist, and at others, emblematic of a phenomenon far more complex than ageism (Gonyea & Hudson, 2020).

A large number of videos tapped into the anxieties of younger people about a generation deemed to be obstructing progressive goals, lending credence to prior scholarship on how tension between generations may be a result of perceived differences in values, sometimes even before any interaction takes place. Similarly, hostility toward Baby Boomers was shown to be linked to perceptions that they have hoarded the nation’s wealth—an unsurprising finding given the tendency of the media to sensationalize Baby Boomers as wealthy and demanding consumers who perpetually steal resources from the young (Bristow, 2019; Sinclair, 2015).

Younger people’s attempts to retaliate against perceived generational differences in values were at times attended by ageist commentary, hinting at how generational and age stereotypes may overlap with each other. Ageist content also surfaced in videos where users shared about their experiences of having been antagonized by their older counterparts on the basis of the former’s generational membership. Past literature has indicated that there exists a time-honored tendency for humanity to denigrate younger generations, a situation encapsulated by the common refrain “kids these days” (Protzko & Schooler, 2019). Consistent with social identity theory—central to which is the idea that individuals will attempt to defend the value of their social identity when it is directly attacked by the outgroup (Branscombe et al., 1999)—the derogation of older adults may constitute a defence strategy for the restoration of collective self-esteem among younger people (Branscombe et al., 1999).

In the majority of the videos sampled, younger people discussed negative encounters they had with individuals who happened to be older. The use of the hashtags #OkBoomer or #Boomer in these instances reflects how certain people attribute these negative experiences to encounters with “Boomers,” implying a belief that such behaviors are defects exclusive to this group. This finding parallels earlier research that argues that prior negative contact may intensify feelings of prejudice as well as the propensity to avoid members of a particular group (Barlow et al., 2012; Hayward et al., 2017).

Having undergone various iterations, the “Karen” meme initially originated as a critique of difficult and entitled customers and over time developed into an epithet signifying white supremacy (Negra & Leyda, 2021). The emergence of the “Karen” meme as a theme in our findings was unexpected given that “Karens” are frequently thought to be middle-aged (Greenspan, 2020). Nevertheless, in 2021, a video documenting a heated exchange between a young couple and a lady dubbed “Boomer Karen” went viral on TikTok (Coë, 2021). Our results therefore illustrate that “Karens” may be regarded by younger people as a subcategory of “Baby Boomers,” or “Baby Boomers” as a subcategory of “Karens.”

Whether the “Karen” meme counts as an example of gendered ageism remains a point of contention in mainstream discourse. Some register its ageist uses (Freeman, 2020), situating the meme in a broader context that acknowledges the reality of double jeopardy, whereby the interaction between ageism and sexism worsens the stigma against older women (Krekula et al., 2018). Others stand by the historical origins of the meme, contending that the issue it brings up is not so much age—particularly considering the rise of the “Becky” meme, Karen’s younger equivalent (Applewhite, 2020; Dictionary.com, 2020)—as it is the idea that some women are weaponizing their White privilege against members of the Black community in America (Wilkes, 2020). Further research is needed to clarify if the meme is coopted by individuals in a manner that reflects or perpetuates gendered ageism.

### Implications, Limitations, and Future Directions

Considering its ability to shape public opinion (Gerbner et al., 2002), the media should avoid framing social problems in terms of generations and pitting older and younger groups against each other. Although generations may account for some degree of shared experience, experience is more realistically informed by other axes of inequality such as race, gender, and class (Applewhite, 2021). A fixation with generational differences only deflects from deeper issues of power and privilege which cut across all generations (Applewhite, 2021; Cohen, 2021).

Similar to past social media analyses (Levy et al., 2014; Skipper & Rose, 2021), this study found some evidence of ageism among younger people. Ageism has grave implications for not just the aging cohort but everyone who will enter old age. According to stereotype embodiment theory, ageist beliefs are solidified early in one’s life and assimilated into one’s self-concept in a way that predicts health outcomes in later life (Levy, 2009). Hence, it is imperative to educate younger people about the pernicious effects of ageism (Levy, 2018). At the same time, our results underscore the need to engage with evidence that young people today are indeed grappling with a distinct set of problems that ought not to be overlooked or even dismissed (Applewhite, 2019). For instance, statistics have shown that young people face bleaker economic prospects than their predecessors (Cannon & Kendig, 2018), a situation that has worsened amid the ongoing COVID-19 crisis (Parker & Igielnik, 2020).

Additionally, policymakers should consider that negative evaluations of older people may be influenced by a history of negative contact (Ng et al., 2021c). Existing efforts to reframe aging (Ng & Indran, 2021c, 2022a, 2022b; Sweetland et al., 2017) and to promote positive intergenerational contact should ensure that younger people are exposed to counter-stereotypical exemplars, such as older adults who are warm and amicable. This will aid in mitigating implicit bias against older persons (Busso et al., 2019). Previous studies have uncovered the promising finding that the damaging consequences of prior negative contact may be tempered and eventually eradicated by a greater supply of positive contact experiences (Paolini et al., 2010).

Rather than focusing solely on eliminating stereotypes, it is important to question the veracity of these stereotypes when evinced actual evidence. The reality is that Baby Boomers, as with all other generations, are significantly more heterogeneous than is acknowledged. Specific values or beliefs should not be pinned on any one generation. Likewise, differences in beliefs should be conveyed through open communication to resolve conflict (Derr, 1972). Ultimately, social issues must be understood as the product of oppressive structures rather than individual generations (Applewhite, 2021).

Despite the reputation of TikTok as the territory of younger people, an increasing number of older adults have been making the rounds on the platform especially since the pandemic (Nakamura, 2020; Pfeffer, 2020). The presence of older persons on social media should be encouraged on a larger scale to facilitate dialogue across generations, particularly in view of evidence highlighting the potential for online venues to foster generational connection (Sipocz et al., 2021). Furthermore, unlike other social media platforms, TikTok is unique in that it has features that encourage interaction between members of the TikTok community. For instance, the “duet” feature allows individuals to position another user’s preexisting video with a new clip and is typically used by those who wish to “reply” to or “comment” on a particular video (Hutchison, 2020). Similarly, the “stitch” feature enables individuals to reuse snippets of other users’ videos and is often used to engage in dialogue (Hutchison, 2020). A recently introduced feature, the “Q&A” (Question and Answer), allows creators to respond to their followers’ questions in the form of a text or video (Porter, 2021). These features could be employed in a way that exposes both older and younger people to each other’s thoughts and views.

Moving forward, it is critical to consider how age and generational stereotypes may meld with each other. While researchers and practitioners often focus on the impact of negative age stereotypes on the self-concepts of older adults (Levy, 2009), little is known about how generational stereotypes may affect self-evaluations. Evidence suggests that one’s generational identity may buffer the impact of negative age stereotypes on one’s self-concept by providing one with a sense of positive self-regard and generativity (Weiss & Zhang, 2020). It therefore stands to reason that negative generational stereotypes will likely exacerbate the effects of negative age stereotypes on older adults’ self-evaluations—a promising area for future research.

This study has several limitations. First, it is probable that there were inaccuracies in identifying the age of each user in our use of a facial recognition software (Fiallos et al., 2021; Serrano et al., 2020). Hence, not all the videos analyzed may have been created by individuals who were part of our desired age range. Second, we included only posts that were in English or had captions in English. This represented a broader limitation with the study adopting a Western-centric perspective as most videos analyzed were created by users based in the West. Resentment toward older adults among individuals from Asian societies—where attitudes toward older persons are shaped by norms related to filial piety (Sung, 2001)—is likely to assume a different nature. Future studies could consider analyzing videos from platforms such as Douyin, which has a similarly large following in China, where TikTok is not available.

Third, because we did not have information regarding users’ intentions in uploading the videos analyzed, the insights were derived solely from the content of each video and shaped by the researchers’ own deductions. To minimize potential bias, the videos were rated by two researchers and evidenced high interrater reliability. Nevertheless, in view of the rising popularity of TikTok and its power to shape public perceptions, we encourage more TikTok-related research on the topic that could include psychometrics (Ng, Allore et al., 2020; Ng & Levy, 2018; Ng & Rayner, 2010), big data techniques (Giest & Ng, 2018; Ng, 2018; Ng, Lim et al., 2020; Ng & Tan, 2021a) used to analyze news media (Ng, 2021a, 2021b) across cultures (Ng et al., 2021a, 2021b; Ng & Indran, 2021a, 2021b; Ng & Tan, 2021b) and time (Ng & Chow, 2021).

Despite these limitations, our study contributes to the field of gerontology by expounding on how Baby Boomers are discursively constructed on a platform monopolized by younger people. In doing so, we managed to tease out the nuances inherent in younger people’s usage of the hashtags #OkBoomer and #Boomer. Further research in this area is needed to understand how to strengthen intergenerational relationships.

To conclude, this study explored the mechanisms linked to hostility toward Baby Boomers through an analysis of videos created by younger people on TikTok. Now more than ever it is crucial that older and younger people find common ground. Society is ultimately well served by intergenerational interaction. A preoccupation with generational differences only sidetracks from the real issues that divide society, and the coalitions needed to create a better future.

## Data Availability

Data are publicly available at https://www.tiktok.com/
